# Systemic Inflammation and Gastrointestinal Complications in HIV Patients: A Cross-Sectional Study on the Role of Type II Diabetes

**DOI:** 10.3390/pathogens14010034

**Published:** 2025-01-06

**Authors:** Madalina-Ianca Suba, Bogdan Hogea, Ahmed Abu-Awwad, Daniela Gurgus, Roxana Folescu, Madalina-Otilia Timircan, Simona-Alina Abu-Awwad

**Affiliations:** 1 Doctoral School, “Victor Babes” University of Medicine and Pharmacy, Eftimie Murgu Square, No. 2, 300041 Timisoara, Romania; madalina.suba@umft.ro; 2 Dr. Victor Babes, Infectious Diseases and Pneumophthisiology Hospital Timisoara, 300310 Timisoara, Romania; 3 Department XV—Discipline of Orthopedics-Traumatology, “Victor Babes” University of Medicine and Pharmacy, 300041 Timisoara, Romania; hogea.bogdan@umft.ro (B.H.); ahm.abuawwad@umft.ro (A.A.-A.); 4 Research Center University Professor Doctor Teodor Sora, “Victor Babes” University of Medicine and Pharmacy, 300041 Timisoara, Romania; 5 Department of Balneology, Medical Recovery and Rheumatology, Family Discipline, Center for Preventive Medicine, “Victor Babeș” University of Medicine and Pharmacy, 300041 Timisoara, Romania; folescu.roxana@umft.ro; 6 Ist Clinic of Obstetrics and Gynecology, “Pius Brinzeu” County Clinical Emergency Hospital, 300723 Timisoara, Romania; madalina-otilia.timircan@umft.ro (M.-O.T.); alina.abuawwad@umft.ro (S.-A.A.-A.); 7 Department of Obstetrics and Gynecology, Faculty of Medicine, “Victor Babes” University of Medicine and Pharmacy, 300041 Timisoara, Romania

**Keywords:** HIV, antiretroviral therapy, type II diabetes, inflammatory biomarkers, gastrointestinal side effects, chronic inflammation

## Abstract

(1) Background: This study aimed to assess the association between inflammatory biomarkers and gastrointestinal side effects in HIV-positive patients on antiretroviral therapy (ART), with a specific focus on the impact of type II diabetes. (2) Methods: A total of 320 participants were divided into three groups: 120 HIV-positive without diabetes, 80 HIV-positive with type II diabetes, and 120 controls. Biomarkers such as CRP, IL-6, and TNF-α, along with gastrointestinal symptoms, were measured before and six months after ART. (3) Results: HIV-positive patients with type II diabetes exhibited significantly elevated levels of inflammatory markers and experienced more frequent gastrointestinal side effects, particularly nausea and diarrhea. (4) Conclusions: Type II diabetes significantly worsens inflammation and gastrointestinal side effects in HIV patients on ART, suggesting the need for tailored treatment approaches.

## 1. Introduction

The introduction of antiretroviral therapy (ART) has significantly extended the life expectancy of individuals infected with the Human Immunodeficiency Virus (HIV), transforming a once-deadly infection into a chronic, manageable disease [1]. Highly efficient and well-tolerated combination ART regimens have made this possible. However, despite these advancements, HIV infection remains a significant public health challenge. Lifelong treatment is required to suppress viral replication and prevent opportunistic infections, as no definitive cure exists [2]. Long-term ART, while effective in controlling HIV, has been associated with various complications, including cardiovascular disease, osteoporosis, and cognitive decline—conditions often linked to aging [3]. One of the contributing factors to these complications is chronic inflammation and immune activation, which persist despite viral suppression.

Chronic inflammation and immune activation are significant concerns in people living with HIV (PLHIV). These processes drive premature immunosenescence, which accelerates aging in this population. In this context, inflammatory biomarkers play a crucial role in identifying and managing non-AIDS-related complications [4]. ART has revolutionized HIV management, and biomarkers such as HIV-1 RNA have been essential in assessing treatment efficacy. However, the persistence of inflammation, particularly in the gastrointestinal (GI) system, remains a challenge for many PLHIV.

The gastrointestinal tract, especially the gut-associated lymphoid tissue (GALT), is critical in HIV pathogenesis. During acute HIV infection, the virus rapidly depletes CD4+ T cells in the GALT, leading to significant epithelial damage and the disruption of intestinal homeostasis. Even with long-term ART and the near-normal restoration of CD4+ cell counts, chronic low-grade inflammation persists in the GI system, contributing to enteropathy and other gastrointestinal side effects. These issues are compounded by the toxicity of some ART drugs, which can exacerbate GI symptoms such as nausea, diarrhea, and abdominal discomfort [5].

In recent years, there has been increased interest in understanding the role of inflammatory biomarkers in predicting ART-associated side effects, particularly those affecting the gastrointestinal system. Biomarkers such as C-reactive protein (CRP), interleukin-6 (IL-6), and tumor necrosis factor-alpha (TNF-α) have been shown to correlate with systemic inflammation and immune dysfunction, which may play a role in the development of GI side effects [6]. However, the situation is further complicated by the presence of coexisting conditions, particularly type II diabetes, which is common among people living with HIV [7].

Type II diabetes in HIV-infected patients presents unique clinical challenges, as it is known to exacerbate systemic inflammation, leading to increased morbidity in individuals with both conditions [8]. The dual burden of HIV and type II diabetes can lead to more severe gastrointestinal issues and heightened levels of inflammatory biomarkers compared to patients infected only with HIV. Understanding the interaction between HIV, ART, and type II diabetes concerning inflammation and GI toxicity is crucial for optimizing treatment strategies in these populations.

This study aims to explore the role of inflammatory biomarkers in predicting gastrointestinal side effects in HIV-positive patients undergoing modern antiretroviral therapy, with a focus on the impact of type II diabetes. By examining the levels of critical cytokines and markers of inflammation, this research seeks to identify predictors of GI discomfort in ART patients, particularly those with diabetes. Understanding these associations may help clinicians mitigate the side effects of therapy, improve treatment adherence, and ultimately enhance the quality of life for individuals living with both HIV and type II diabetes.

## 2. Materials and Methods

### 2.1. Study Design and Population

This study was designed as a cross-sectional, observational analysis to explore the association between inflammatory biomarkers and gastrointestinal side effects in HIV-positive individuals undergoing ART. The study population was divided into three distinct groups. The first group (120 patients), serving as the control group, included individuals who were HIV-negative and diabetes-free, providing baseline data for comparison on inflammatory biomarkers and gastrointestinal health. The second group (120 patients) consisted of HIV-positive patients undergoing antiretroviral therapy (ART) without type II diabetes, allowing for the analysis of ART-related inflammatory responses and gastrointestinal side effects in the absence of diabetes. The third group (80 patients) included HIV-positive patients who also had diabetes and were undergoing ART, facilitating the assessment of how type II diabetes influences both inflammation and gastrointestinal complications compared to the other two groups. We chose to focus solely on type II diabetes for this study because it is significantly more prevalent in the general population of HIV-positive individuals, and its association with chronic low-grade inflammation provides a more relevant context for examining the interaction between diabetes, antiretroviral therapy, and gastrointestinal side effects.

In this study, HIV patients were exclusively treated with standardized regimens of new-generation ART to reduce variability among participants and minimize drug-related toxic effects. Most patients received regimens based on integrase strand transfer inhibitors (INSTIs), such as dolutegravir, combined with nucleoside reverse transcriptase inhibitors (NRTIs), such as tenofovir alafenamide (TAF) and emtricitabine. These regimens were chosen for their excellent safety profiles and high efficacy, allowing us to minimize the confounding effects of drug-specific toxicities on gastrointestinal symptoms and inflammatory markers. All patients diagnosed with HIV were in their seventh month (after completing six months) of ART at the time their gastrointestinal symptoms were assessed, and inflammatory markers were re-measured. The period of six complete months was chosen as it represents a critical stage where the effects of ART typically stabilize, including viral suppression and partial immune recovery, minimizing variability from initial immune responses. This uniform timeframe ensured consistent exposure to ART across all participants, enabling more accurate comparisons between groups. Additionally, six months is an appropriate period to observe changes in inflammatory markers while still capturing ongoing inflammation influenced by factors such as type II diabetes.

For patients with type II diabetes, the therapeutic approach included antidiabetic medications with a low risk of gastrointestinal side effects, avoiding the use of metformin due to its potential to exacerbate gastrointestinal symptoms. Instead, medications such as SGLT-2 inhibitors (e.g., empagliflozin) and DPP-4 inhibitors (e.g., sitagliptin) were frequently used due to their favorable safety profiles. SGLT-2 inhibitors also provide additional benefits, including reduced cardiovascular risk and renal protection, without significantly affecting the gastrointestinal tract. Patients included in the ART + diabetes group had a preexisting diagnosis of type II diabetes prior to initiating antiretroviral therapy (ART). This approach ensured the evaluation of systemic inflammation and gastrointestinal symptoms in the context of established diabetes, eliminating the variability associated with diabetes induced by ART. By focusing on patients with diabetes diagnosed before ART, this study aimed to specifically analyze the interaction between this comorbidity and ART-related effects.

Both ART and antidiabetic regimens were meticulously documented and included in the statistical analyses to account for their potential influence on inflammatory markers and gastrointestinal symptoms. This standardized approach ensures a more precise assessment of the relationship between systemic inflammation, ART, and associated comorbidities while minimizing the confounding effects of medication-related toxicities.

The severity of gastrointestinal symptoms, including nausea, diarrhea, abdominal pain, and bloating, was assessed and classified using the Common Terminology Criteria for Adverse Events (CTCAE, version 5.0). According to this framework, symptoms were graded as follows: Grade 1 (mild), indicating minimal impact on daily activities; Grade 2 (moderate), defined as persistent or recurrent symptoms that interfere with daily activities and may require symptomatic intervention; and Grade 3 (severe), characterized by significant disruption of daily life and necessitating active medical management. For this study, clinical evaluations corroborated patient-reported outcomes to ensure consistency and objectivity in symptom grading. The majority of gastrointestinal symptoms were classified as Grade 2, with a subset of patients, particularly those with coexisting diabetes mellitus, exhibiting Grade 3 severity. This grading system allowed for a standardized and reproducible assessment of symptom severity across all study groups, providing a robust framework for evaluating the interplay between antiretroviral therapy, diabetes, and gastrointestinal side effects.

Demographic and clinical factors, such as age, gender, body mass index (BMI), and comorbidities, were considered across all groups to ensure robust and accurate comparisons.

Participants were selected from a large urban health center specializing in HIV care and management. The study was conducted at the “Dr. Victor Babeș Clinical Hospital for Infectious Diseases and Pneumophthisiology” in Timișoara, Romania. Two hundred forty people were enrolled in the study, with 80 HIV-positive patients who also had diabetes (ART + diabetes group), 120 patients in an ART group, and 120 in a control group. Biological data were collected at the initiation of treatment and again six months later, with patients being enrolled over a 10-year period from January 2013 to January 2023.

### 2.2. Inclusion and Exclusion Criteria

To ensure the study’s relevance and rigor, strict inclusion criteria were established for the patients involved in the research. These criteria aim to select patients with suitable profiles for assessing this study.

Inclusion criteria:

- Participants with a confirmed diagnosis of HIV infection and type II diabetes.
- Patients 18 years of age or older.
- Patients in the ART group must be on antiretroviral therapy for six months.
- Omnivorous patients who consume meat 5 to 7 days per week, given that dietary habits, including meat intake, can significantly influence inflammatory processes within the body.
- Patients who agree to participate in the study and agree to sign the informed consent.
- Participants who can comply with study requirements, including reporting to follow-up visits and collection of biological samples.
- Clinically stable participants without signs of acute infections or other conditions that require urgent treatment and interfere with the state of health.
- Patients have not been under treatment with immunosuppressants or anti-inflammatory drugs in the last year, as they could influence the level of inflammatory biomarkers.
- Participants without known allergies to the substances that make up the ART treatments.
- Participants must have the ability to understand and communicate effectively in Romanian to complete questionnaires accurately, provide reliable information during interviews, and fully comply with study requirements.

Exclusion criteria:

- Patients with diagnosed autoimmune diseases such as systemic lupus erythematosus or multiple sclerosis are excluded because of the potential impact on inflammation and immune response.
- Individuals who have reported a significant change in diet in the past 3 months.
- Patients who have been recently diagnosed with severe non-HIV infections such as tuberculosis or severe pneumonia.
- Patients with specific comorbidities that potentially impact gastrointestinal symptoms may be excluded from the study, as these conditions could confound the results and complicate the interpretation of the findings.
- Patients who have used long-term corticosteroids for other conditions, as these drugs can suppress the inflammatory response.
- Patients with kidney diseases that require specialized treatment or dialysis.
- Patients with significant liver damage could complicate the interpretation of inflammatory markers.
- Patients with cardiovascular diseases.
- Patients who are currently undergoing treatment for cancer, including chemotherapy or radiotherapy, due to the potential impact on the immune system.
- Patients who are known to be actively using illicit drugs or excessive alcohol are excluded, as these behaviors may affect study results.
- Patients who have had severe adverse reactions to a wide range of drugs are excluded to avoid the risk of complications during the study.
- Patients who have recently undergone immunomodulatory treatments (TNF inhibitors, interferons) are excluded to prevent influences on systemic inflammation.
- Patients with severe cognitive impairment or psychiatric conditions that could affect the ability to understand or comply with the study protocol are excluded.

### 2.3. Statistical Analysis

The statistical analysis was performed using MedCalc Statistical Software (Version number 20.0), leveraging its specialized functionalities for hypothesis testing, regression modeling, and data visualization. Continuous variables were summarized as means with standard deviations, while categorical variables were expressed as frequencies and percentages. The selection of statistical methods was guided by the distribution and nature of the data, as determined through the rigorous testing of assumptions.

To assess the distribution of continuous variables, normality was tested using the Shapiro–Wilk test, while the homogeneity of variances was evaluated using Levene’s test. For variables such as viral load, which exhibited a highly skewed distribution, a Log10 transformation was applied to achieve normality. Post-transformation, normality, and homoscedasticity were reassessed to ensure the validity of subsequent statistical analyses. For variables that failed these assumptions even after transformation (e.g., biomarkers with high variability), non-parametric methods such as the Kruskal–Wallis test were employed as alternatives to the ANOVA.

For continuous variables meeting parametric assumptions, a one-way ANOVA was used to compare means across the three groups, followed by post hoc analyses to identify significant pairwise differences. Categorical variables were analyzed using the chi-square test to evaluate proportion differences between groups. For variables with small, expected cell counts, Fisher’s exact test was used to ensure accurate results.

Relationships between inflammatory biomarkers and gastrointestinal symptoms were examined using Pearson correlation coefficients for linear associations. Spearman’s rank correlation was used as a robust, non-parametric alternative for variables or relationships that violated linearity or normality assumptions.

Multivariate logistic regression was utilized to identify independent predictors of gastrointestinal symptoms, with the dependent variable being a binary outcome indicating the presence or absence of gastrointestinal symptoms 6 months after antiretroviral therapy (ART). Predictor variables, including inflammatory biomarkers (e.g., CRP, IL-6, TNF-α), age, and BMI, were chosen based on their clinical relevance and supporting evidence from previous studies. Collinearity diagnostics were conducted to ensure the model’s validity, confirming the independence of the predictor variables. Additionally, Bonferroni corrections were applied to control for the family-wise error rate, and post hoc multiple comparisons were conducted where appropriate.

Missing data were addressed through appropriate methods: listwise deletion was used for variables with a small percentage of missing values (<5%). At the same time, multiple imputation was applied for variables with more substantial gaps to ensure robust analyses.

A significance threshold of *p* < 0.05 was used for all tests, except where adjustments for multiple comparisons were necessary. The analytical approach was designed to account for potential confounding variables and ensure reproducibility and rigor in interpreting results.

By systematically applying these statistical methods, the study ensured that the findings were both reliable and scientifically robust, offering valid insights into the relationships between inflammatory biomarkers, gastrointestinal outcomes, and group characteristics.

### 2.4. Ethical Considerations

The patient’s medical records were securely maintained in a database compliant with data protection laws. Access to these records was granted only after obtaining informed consent from the patients, safeguarding their confidentiality, and ensuring their rights were respected.

The procedures carried out in this research adhered strictly to the guidelines set by the relevant human experimentation committee at both institutional and national levels. They were also aligned with the principles of the Helsinki Declaration of 1975, as revised in 2013. Informed consent was obtained from each patient included in the study, ensuring they were thoroughly informed about the nature and scope of the research.

This research was approved by the hospital’s Ethics Committee, with approval number 8947/28 September 2018. This approval verified that the study adhered to all pertinent ethical guidelines, ensuring the protection and respect of all human participants involved in the research.

## 3. Results

Table 1 presents the demographic and clinical characteristics of the study participants, comparing the control group (CG), the ART group, and the ART + type II diabetes group. Key parameters include age, gender, body mass index (BMI) and CD4+ T cell count. The similar distribution of age and gender across the groups enhances the study populations’ comparability, minimizing the confounding variables’ potential influence. The significant difference observed in CD4+ T cell counts between the groups highlights the immunological compromise in the ART and ART + type II diabetes groups compared to the control, reflecting the chronic immune activation and viral burden associated with HIV infection. By ensuring balanced demographic and clinical characteristics across the groups, the study enables a more accurate assessment of the relationship between inflammatory biomarkers and gastrointestinal outcomes while controlling for underlying variables. No significant differences were observed in the mean BMI values across the three groups, indicating that the impact of BMI does not influence the study’s results, further reinforcing the relevance of the variables under investigation.

Table 2 illustrates the differences in inflammatory biomarker levels before and after 6 months of ART, highlighting variations among the control group, ART group, and ART + type II diabetes group. Before treatment, biomarker levels were significantly higher in the ART groups, with the highest values observed in the ART + diabetes group, indicating more severe systemic inflammation associated with both therapy and the presence of diabetes. After 6 months of ART, significant reductions in inflammatory biomarkers were observed in the treated groups. The ART group showed consistent and statistically significant reductions in all analyzed biomarkers, reflecting an overall decrease in systemic inflammation due to treatment. In the ART + diabetes group, reductions were significant for most biomarkers but less pronounced compared to the non-diabetic group, suggesting that the presence of diabetes may influence the response to therapy. In contrast, in the control group, biomarker levels remained stable over the 6-month period, with no significant changes, as expected in the absence of ART. The analysis of *p* values confirms that the differences were significant in the treated groups but not in the control group. Overall, the data highlight the beneficial effects of ART in reducing inflammation but also emphasize the impact of diabetes in limiting these effects.

The analysis of gastrointestinal adverse reactions across the control group, ART group, and ART + type II diabetes group demonstrates significant differences in symptoms such as nausea, diarrhea, abdominal pain, and bloating 6 months after treatment (Table 3). A markedly higher prevalence of these side effects was observed in the ART and ART + type II diabetes groups compared to the control group, with statistically significant *p* values (<0.0001 for all symptoms). These results suggest a strong association between antiretroviral therapy, especially in the presence of type II diabetes, and gastrointestinal side effects. Such adverse reactions may negatively impact treatment adherence and overall quality of life for patients, underscoring the need for proactive management strategies to mitigate these effects and ensure optimal therapeutic outcomes.

The correlations presented in Table 4 highlight the relationships between inflammatory biomarkers (e.g., CRP, IL-6, TNF-α, fibrinogen) and gastrointestinal symptoms such as nausea, diarrhea, abdominal pain, and bloating observed 6 months after ART. The Pearson correlation coefficients (r) quantify the strength of these associations, with all marked values indicating statistical significance at the *p* < 0.01 level. Strong positive correlations, such as TNF-α with nausea (r = 0.68) and diarrhea (r = 0.63), and CRP with nausea (r = 0.65) and diarrhea (r = 0.60), suggest that persistent systemic inflammation is a critical factor in the development of these gastrointestinal side effects, particularly in patients with HIV and type II diabetes. The stars (*) signify these significant associations.

The results presented in Table 5 derive from a multivariate logistic regression analysis aimed at identifying key predictors associated with the occurrence of gastrointestinal side effects in patients 6 months after starting antiretroviral therapy (ART). The study evaluated several inflammatory biomarkers, including CRP, IL-6, TNF-α, and fibrinogen, along with other variables like IL-1β, IFN-γ, D-dimer, age, and BMI. For each parameter, the table includes the Odds Ratio (OR), 95% confidence intervals (CIs), and *p* values to assess the statistical significance of these associations.

The findings demonstrate that elevated levels of CRP, IL-6, TNF-α, and fibrinogen are all significant predictors of gastrointestinal symptoms, as indicated by their high OR values and narrow confidence intervals. For instance, TNF-α exhibited an OR of 1.82 (95% CI: 1.45–2.31, *p* < 0.0001), underscoring its strong association with gastrointestinal side effects. Similarly, markers such as CRP (OR 1.75) and IL-6 (OR 1.68) also show a significant predictive value for the occurrence of these symptoms.

In addition, IL-1β, IFN-γ, and D-dimer emerged as independent predictors of gastrointestinal complications, reinforcing the critical role of systemic inflammation in developing these adverse effects, particularly in patients with HIV and type II diabetes.

## 4. Discussion

The results of this study provide valuable insights into the complex relationship between systemic inflammation, gastrointestinal side effects, and the impact of antiretroviral therapy (ART) in HIV-positive patients, particularly those with type II diabetes. By analyzing inflammatory biomarkers and their association with gastrointestinal symptoms, this research not only aligns with the existing literature but also highlights the urgent need for tailored therapeutic strategies to optimize patient outcomes.

Previous studies have demonstrated the persistence of systemic inflammation in HIV-positive patients undergoing ART, even with effective viral suppression [9]. Our results corroborate these findings [10], as both the ART and ART + type II diabetes groups exhibited consistently elevated levels of inflammatory biomarkers, including CRP, IL-6, TNF-α, and fibrinogen, compared to the control group. Similar to other observations, these biomarkers remained elevated despite significant reductions after six months of ART. This persistent inflammation aligns with other findings linking chronic immune activation to non-AIDS comorbidities, such as cardiovascular disease [11], diabetes [12], and neurocognitive disorders [13].

The ART + type II diabetes group demonstrated less pronounced reductions in biomarker levels compared to the ART group, emphasizing the cumulative effects of diabetes on systemic inflammation. This observation aligns with some findings that reported that diabetes exacerbates inflammation through mechanisms such as oxidative stress and advanced glycation end-products [14,15,16]. Our results extend these findings, highlighting the double inflammatory burden experienced by patients with both HIV and type II diabetes.

A significant contribution of this study is the demonstrated association between elevated inflammatory biomarkers and gastrointestinal symptoms. Biomarkers such as TNF-α, CRP, and IL-6 showed strong correlations with nausea, diarrhea, abdominal pain, and bloating, with the most severe symptoms observed in the ART + type II diabetes group. These findings support previous studies identifying inflammation as a central factor in ART-induced gastrointestinal side effects [17,18]. For instance, TNF-α, a key mediator of the inflammatory cascade, is implicated in increasing intestinal permeability and microbial translocation, perpetuating chronic immune activation and contributing to gastrointestinal dysfunction [19].

Mechanistically, HIV-induced damage to gut-associated lymphoid tissue (GALT) compromises intestinal epithelial integrity, allowing microbial products to enter systemic circulation and exacerbate immune activation [5]. Diabetes amplifies these effects by promoting oxidative stress and gut barrier dysfunction [20]. Our findings align with studies suggesting that ART-associated intestinal dysregulation, combined with diabetes-specific inflammation, exacerbates gastrointestinal symptoms [21].

The results underscore the limitations of standardized ART regimens in managing the dual burden of HIV and diabetes. While new-generation ART regimens, based on INSTIs and NRTIs, are associated with fewer toxicities, their effectiveness in reducing systemic inflammation appears compromised in patients with comorbidities. This highlights the need for personalized therapeutic strategies that address the unique inflammatory and metabolic challenges of this population [22].

Personalized ART regimens that prioritize gut health and reduce inflammatory side effects are essential. Strategies such as selecting ART combinations with minimal gastrointestinal toxicity or adjusting dosages based on inflammatory profiles could help reduce symptom severity. For example, transitioning patients with severe gastrointestinal symptoms to alternative ART regimens with better safety profiles may mitigate side effects while maintaining viral suppression [23].

Incorporating adjuvant therapies targeting systemic inflammation represents another promising avenue. Agents such as TNF-α inhibitors or IL-6 receptor blockers could directly modulate inflammatory pathways contributing to gastrointestinal symptoms. Gut-specific interventions, including probiotics, prebiotics, and immunomodulatory agents, may help restore epithelial integrity and microbiome balance, reducing both inflammation and gastrointestinal distress [24].

Given the exacerbating effects of diabetes on inflammation and gastrointestinal symptoms, glycemic control is crucial. Using antidiabetic agents such as SGLT-2 and DPP-4 inhibitors, which have low gastrointestinal toxicity and anti-inflammatory properties, supports the need for personalized treatment. Avoiding medications like metformin in patients predisposed to gastrointestinal symptoms further emphasizes the importance of individualized care [8].

The persistence of inflammatory biomarkers after six months of ART highlights the need for routine monitoring to identify patients at higher risk of inflammatory complications. Regular assessments of biomarkers such as CRP, IL-6, TNF-α, and fibrinogen can guide timely interventions and optimize therapeutic strategies. Implementing predictive models based on these biomarkers may help identify individuals who would benefit most from tailored treatment adjustments [22].

Addressing systemic inflammation and its gastrointestinal consequences is essential to improving ART adherence and quality of life in HIV-positive patients. Severe gastrointestinal symptoms, in particular, significantly impact daily activities and can lead to suboptimal ART adherence, jeopardizing long-term viral suppression and overall patient outcomes.

Future research directions should include longitudinal studies spanning 12–24 months to evaluate the long-term impact of systemic inflammation on gastrointestinal symptoms and comorbidities, providing opportunities to identify optimal timing for therapeutic interventions. Investigating biomarkers as predictive tools for gastrointestinal and systemic complications, alongside establishing critical thresholds for guiding early interventions, is a priority. Additionally, clinical studies exploring the efficacy of adjuvant therapies, such as probiotics and anti-inflammatory agents, could help reduce inflammation and improve gastrointestinal outcomes. Integrating precision medicine approaches, including genetic profiling and individualized ART regimens, could optimize care by tailoring treatments to the specific needs of patients based on genetic and metabolic factors.

A significant and innovative contribution of this study lies in its integrated evaluation of inflammatory biomarkers and gastrointestinal symptoms in HIV-positive patients, with a special focus on the influence of type II diabetes. While many studies have separately investigated ART-associated inflammation or gastrointestinal side effects, our research is among the few to explore the complex interplay between these variables in a comparative framework that includes both HIV-positive patients without diabetes and those with type II diabetes. Furthermore, the use of standardized ART regimens based on new-generation medications minimizes variability and allows for a clearer interpretation of relationships between inflammation, comorbidities, and symptoms. The uniform six-month reevaluation period adds a unique methodological dimension, capturing ART effects after treatment stabilization. This approach not only provides a detailed perspective on inflammatory and gastrointestinal processes but also identifies key intervention points, underscoring the need for personalized therapeutic strategies, an aspect insufficiently explored in the existing literature.

### Strengths and Limitations

This study offers several notable strengths. Firstly, it provides new insights into the correlation between elevated inflammatory biomarkers—such as CRP, IL-6, TNF-α, and fibrinogen—and gastrointestinal symptoms in HIV-positive patients on ART, particularly those with type II diabetes. This novel association enriches the existing literature on HIV treatment and its side effects, emphasizing the role of systemic inflammation in gastrointestinal dysfunction. The longitudinal design further enhanced the study’s strength, which involved collecting inflammatory biomarkers both before and 6 months after ART initiation. This allowed for a dynamic evaluation of how systemic inflammation evolves with treatment and how it may contribute to gastrointestinal symptoms over time. Secondly, the comprehensive analysis of a broad range of biomarkers, including D-dimer and IL-1β, allows for a detailed evaluation of the mechanisms behind ART-related side effects, particularly in patients with comorbid conditions like type II diabetes. This thorough biomarker profiling enhances the robustness of the findings and provides a more nuanced understanding of the interplay between inflammation, ART, and gastrointestinal outcomes. Lastly, the study holds significant clinical relevance by highlighting the need for personalized treatment approaches, particularly for patients with elevated inflammatory markers, which could directly impact clinical practice. The improved management of gastrointestinal symptoms could lead to better treatment adherence and enhanced quality of life for HIV-positive patients, especially those with diabetes.

However, this study also has certain limitations. While the longitudinal design offers advantages, the observational nature of this study still limits the ability to establish definitive causality between elevated biomarkers and gastrointestinal symptoms. The six-month timeframe, while critical for observing early ART-related changes, may not fully capture the long-term dynamics of immune recovery, viral suppression, and the establishment of HIV reservoirs in various compartments of the body. Extending the follow-up period in future research would allow a deeper understanding of how systemic inflammation and gastrointestinal outcomes evolve. Additionally, while viral load approached undetectable levels for most participants and CD4+ T cell counts improved, it remains unclear whether all patients achieved their set point levels within six months. These factors underscore the need for more granular analyses of each patient’s stage in their HIV journey.

Expanding this study into a more significant, multi-center cohort would provide more comprehensive insights into how inflammation evolves with ART over extended periods and how it contributes to long-term health outcomes. Additionally, this study was conducted at a single center, which may limit the generalizability of the results to a broader population. A multi-center approach would enhance the external validity of the findings and provide a more diverse patient population for analysis. Lastly, while the study includes 240 participants, future research with larger sample sizes must confirm these findings and improve statistical power, particularly when analyzing subgroups such as those with type II diabetes.

## 5. Conclusions

The findings of this study indicate that, six months after initiating antiretroviral therapy (ART), HIV-positive patients with type II diabetes exhibited significantly elevated levels of systemic inflammatory markers, including CRP, IL-6, TNF-α, and fibrinogen, compared to both HIV-positive patients without diabetes and the control group. This persistent inflammation, more pronounced in those with type II diabetes, suggests that this comorbidity may contribute to chronic immune activation despite effective viral suppression achieved through ART. Additionally, chronic inflammation was associated with a higher prevalence of gastrointestinal side effects, such as nausea, diarrhea, and abdominal pain, which may negatively impact treatment adherence and quality of life.

These results highlight the potential importance of routine inflammation monitoring and personalized treatment adjustments for HIV-positive patients, particularly those with type II diabetes, to address gastrointestinal symptoms and reduce the risk of long-term non-AIDS-related complications. However, further longitudinal studies are needed to confirm these associations and explore the causal mechanisms underlying the observed relationships between systemic inflammation, ART, and gastrointestinal outcomes.

## Figures and Tables

**Table 1:** Demographic and clinical characteristics of study participants.

Characteristic	Control Group (*n* = 120)	ART Group (*n* = 120)	ART + Type II Diabetes Group (*n* = 80)	*p* Value
Age (years) ^(a)^	35 ± 7	36 ± 8	34 ± 6	0.021
Gender (Male/Female) ^(b)^	71 (59.16%)/ 49 (40.83%)	64 (53.33%)/ 56 (46.66%)	41 (51.25%)/ 39 (48.75%)	0.489
CD4 Count (cells/μL) ^(a)^	753 ± 101	458 ± 113	399 ± 243	<0.0001
Viral Load (copies/mL) ^(a)^	Undetectable	10,536 ± 5057	12,355 ± 8454	<0.0001
BMI ^(a)^	26.1 ± 7.9	24.9 ± 4.1	25.2 ± 7	0.413

^(a)^ Standard deviation ± mean; ^(b)^ percentage.

**Table 2:** Biomarker levels before ART initiation.

Biomarker		Control Group (*n* = 120)	ART Group (*n* = 120)	ART + Type II Diabetes Group (*n* = 80)	*p* Value
CRP (mg/L)	Before/ 6 months after ART initiation	1.8 ± 0.9/ 1.8 ± 0.7	2.6 ± 1.1/ 2.2 ± 0.9	4.6 ± 1.2/ 3.9 ± 1.1	<0.001/ <0.001
	*p* Value	>0.999	0.002	0.002	
Interleukin-6 (IL-6) (pg/mL) (a)	Before/ 6 months after ART initiation	4.1 ± 1.0/ 3.9 ± 1.1	5.8 ± 1.9/ 4.8 ± 1.8	8.2 ± 2.1/ 6.8 ± 1.9	<0.001/ <0.001
	*p* Value	0.141	<0.0001	<0.0001	
Tumor necrosis factor-alpha (TNF-α) (pg/mL) (a)	Before/ 6 months after ART initiation	3.5 ± 1.2 3.2 ± 1.0	8.1 ± 2.6 6.5 ± 2.4	13.5 ± 3.1 11.1 ± 2.9	<0.001 <0.001
	*p* Value	0.036	<0.0001	<0.0001	
Fibrinogen (mg/dL) (a)	Before/ 6 months after ART initiation	284 ± 47/ 280 ± 44	461 ± 64/ 430 ± 57	486 ± 63/ 461 ± 55	<0.001/ <0.001
	*p* Value	0.496	<0.001	0.008	
IL-1β (pg/mL) (a)	Before/ 6 months after ART initiation	3.1 ± 1.2/ 2.7 ± 1.0	4.5 ± 1.3/ 4.0 ± 1.2	6.1 ± 1.8/ 5.7 ± 1.5	<0.001/ <0.001
	*p* Value	0.005	0.002	0.128	
IFN-γ (pg/mL) (a)	Before/ 6 months after ART initiation	8.7 ± 2.4/ 8.1 ± 2.1	11.2 ± 3.2/ 9.6 ± 2.7	14.5 ± 3.4/ 13.7 ± 3.0	<0.001/ <0.001
	*p* Value	0.0404	<0.0001	0.116	
D-dimer (mg/L) (a)	Before/ 6 months after ART initiation	0.4 ± 0.3/ 0.4 ± 0.2	0.8 ± 0.6/ 0.7 ± 0.2	1.2 ± 0.6/ 1.1 ± 0.4	<0.001/ <0.001
	*p* Value	>0.999	0.084	0.216	

**Table 3:** Gastrointestinal side effects reported.

Side Effect	Control Group (*n* = 120)	ART Group (*n* = 120)	ARV + Type II Diabetes Group (*n* = 80)	*p* Value
Nausea	11 (9.16%)	50 (41.66%)	45 (56.25%)	<0.001
Diarrhea	5 (4.16%)	44 (36.66%)	43 (53.75%)	<0.001
Abdominal pain	8 (6.66%)	40 (33.33%)	38 (47.5%)	<0.001
Bloating	7 (5.83%)	31 (25.83%)	27 (33.75%)	<0.001

**Table 4:** Correlation between inflammatory biomarkers and gastrointestinal side effects.

Biomarker	Nausea	Diarrhea	Abdominal Pain	Bloating
C-reactive protein (CRP)	0.65 *	0.60 *	0.55 *	0.50 *
Interleukin-6 (IL-6)	0.62 *	0.58 *	0.54 *	0.48 *
Tumor necrosis factor-alpha (TNF-α)	0.68 *	0.63 *	0.57 *	0.52 *
Fibrinogen	0.60 *	0.55 *	0.53 *	0.45 *
IL-1β (pg/mL)	0.39 *	0.36 *	0.34 *	0.37 *
IFN-γ (pg/mL)	0.46 *	0.49 *	0.51 *	0.48 *
D-dimer (mg/L)	0.41 *	0.39 *	0.40 *	0.36 *

* *p* < 0.01.

**Table 5:** Multivariate logistic regression analysis of predictors of gastrointestinal side effects.

Variable	Odds Ratio (OR)	95% Confidence Interval (CI)	*p* Value
CRP	1.75	1.40–2.20	<0.0001
IL-6	1.68	1.37–2.10	<0.0001
TNF-α	1.82	1.45–2.31	<0.0001
Fibrinogen	1.60	1.30–2.00	0.003 *
IL-1β (pg/mL)	1.25	1.06–1.51	0.007 *
IFN-γ (pg/mL)	1.61	1.29–1.84	<0.001 *
D-dimer (mg/L)	1.34	1.14–1.64	<0.001 *
Age	1.05	1.02–1.08	0.010 *
BMI	1.10	1.05–1.20	0.008 *

* Statistically significant results were considered at *p* < 0.05.

## Data Availability

The datasets used and/or analyzed during the current study are available from the first author.

## References

[1] Foka F.E.T., Mufhandu H.T. (2023). Current ARTs, Virologic Failure, and Implications for AIDS Management: A Systematic Review. Viruses.

[2] Günthard H.F., Saag M.S., Benson C.A., del Rio C., Eron J.J., Gallant J.E., Hoy J.F., Mugavero M.J., Sax P.E., Thompson M.A. (2016). Antiretroviral Drugs for Treatment and Prevention of HIV Infection in Adults: 2016 Recommendations of the International Antiviral Society-USA Panel. JAMA.

[3] Jaqua E., Labib W., Danji K. (2022). HIV-Associated Conditions in Older Adults. Cureus.

[4] Justice A.C., Erlandson K.M., Hunt P.W., Landay A., Miotti P., Tracy R.P. (2018). Can Biomarkers Advance HIV Research and Care in the Antiretroviral Therapy Era?. J. Infect. Dis..

[5] Moretti S., Schietroma I., Sberna G., Maggiorella M.T., Sernicola L., Farcomeni S., Giovanetti M., Ciccozzi M., Borsetti A. (2023). HIV-1-Host Interaction in Gut-Associated Lymphoid Tissue (GALT): Effects on Local Environment and Comorbidities. Int. J. Mol. Sci..

[6] Giovannini S., Onder G., Liperoti R., Russo A., Carter C., Capoluongo E., Pahor M., Bernabei R., Landi F. (2011). Interleukin-6, C-reactive protein, and tumor necrosis factor-alpha as predictors of mortality in frail, community-living elderly individuals. J. Am. Geriatr. Soc..

[7] Duncan A.D., Goff L.M., Peters B.S. (2018). Type 2 diabetes prevalence and its risk factors in HIV: A cross-sectional study. PLoS ONE.

[8] Brown T.T., Tassiopoulos K., Bosch R.J., Shikuma C., McComsey G.A. (2010). Association between systemic inflammation and incident diabetes in HIV-infected patients after initiation of antiretroviral therapy. Diabetes Care.

[9] Serlin M.H., Dieterich D. (2008). Gastrointestinal Disorders in HIV. Glob. HIV/AIDS Med..

[10] Lv T., Cao W., Li T. (2021). HIV-Related Immune Activation and Inflammation: Current Understanding and Strategies. J. Immunol. Res..

[11] Henein M.Y., Vancheri S., Longo G., Vancheri F. (2022). The Role of Inflammation in Cardiovascular Disease. Int. J. Mol. Sci..

[12] Tsalamandris S., Antonopoulos A.S., Oikonomou E., Papamikroulis G.A., Vogiatzi G., Papaioannou S., Deftereos S., Tousoulis D. (2019). The Role of Inflammation in Diabetes: Current Concepts and Future Perspectives. Eur. Cardiol..

[13] Sartori A.C., Vance D.E., Slater L.Z., Crowe M. (2012). The impact of inflammation on cognitive function in older adults: Implications for healthcare practice and research. J. Neurosci. Nurs..

[14] Nowotny K., Jung T., Höhn A., Weber D., Grune T. (2015). Advanced Glycation End Products and Oxidative Stress in Type 2 Diabetes Mellitus. Biomolecules.

[15] Prasad K. (2014). Low levels of serum soluble receptors for advanced glycation end products (sRAGE) are associated with increased oxidative stress and inflammation in type 2 diabetes. Int. J. Angiol..

[16] Ménégaut L., Laubriet A., Crespy V., Leleu D., Pilot T., Van Dongen K., de Barros J.P.P., Gautier T., Petit J.M., Thomas C. (2023). Inflammation and oxidative stress markers in type 2 diabetes patients with advanced carotid atherosclerosis. Cardiovasc. Diabetol..

[17] Neuman M.G., Schneider M., Nanau R.M., Parry C. (2012). HIV-Antiretroviral Therapy Induced Liver, Gastrointestinal, and Pancreatic Injury. Int. J. Hepatol..

[18] Sim J.H., Mukerji S.S., Russo S.C., Lo J. (2021). Gastrointestinal Dysfunction and HIV Comorbidities. Curr. HIV/AIDS Rep..

[19] Al-Sadi R., Guo S., Ye D., Rawat M., Ma T.Y. (2016). TNF-α Modulation of Intestinal Tight Junction Permeability Is Mediated by NIK/IKK-α Axis Activation of the Canonical NF-κB Pathway. Am. J. Pathol..

[20] Mostafavi Abdolmaleky H., Zhou J.-R. (2024). Gut Microbiota Dysbiosis, Oxidative Stress, Inflammation, and Epigenetic Alterations in Metabolic Diseases. Antioxidants.

[21] Du Y.T., Rayner C.K., Jones K.L., Talley N.J., Horowitz M. (2018). Gastrointestinal Symptoms in Diabetes: Prevalence, Assessment, Pathogenesis, and Management. Diabetes Care.

[22] Hileman C.O., Funderburg N.T. (2017). Inflammation, immune activation, and antiretroviral therapy in HIV. Curr. HIV/AIDS Rep..

[23] Maritati M., Alessandro T., Zanotta N., Comar M., Bellini T., Sighinolfi L., Contini C. (2020). A comparison between different anti-retroviral therapy regimes on soluble inflammation markers: A pilot study. AIDS Res. Ther..

[24] Humphry N. (2022). Adalimumab, golimumab, and infliximab in immune-mediated inflammatory diseases: A review. EMJ Gastro..

